# Deep learning nomogram based on Gd-EOB-DTPA MRI for predicting early recurrence in hepatocellular carcinoma after hepatectomy

**DOI:** 10.1007/s00330-023-09419-0

**Published:** 2023-02-14

**Authors:** Meng Yan, Xiao Zhang, Bin Zhang, Zhijun Geng, Chuanmiao Xie, Wei Yang, Shuixing Zhang, Zhendong Qi, Ting Lin, Qiying Ke, Xinming Li, Shutong Wang, Xianyue Quan

**Affiliations:** 1 Department of Radiology, The First Affiliated Hospital of Jinan University, No. 613, Huangpu West Road, Tianhe District, Guangzhou, 510627 Guangdong People’s Republic of China; 2 Neusoft Research of Intelligent Healthcare Technology, Co. Ltd., Artificial Intelligence and Clinical Innovation Research, Guangzhou, 510000 Guangdong People’s Republic of China; 3 Department of Medical Imaging, Sun Yat-Sen University Cancer Center, No. 651, Dongfeng East Road, Yuexiu District, Guangzhou, 510060 People’s Republic of China; 4 Guangdong Provincial Key Laboratory of Medical Image Processing, School of Biomedical Engineering, Southern Medical University, No. 1023, Shatai Road, Baiyun District, Guangzhou, 510515 Guangdong People’s Republic of China; 5 Department of Radiology, Zhujiang Hospital, Southern Medical University, No. 253, Industrial Road, Haizhu District, Guangzhou, 510282 People’s Republic of China; 6 Medical Imaging Center, the First Affiliated Hospital of Guangzhou University of Chinese Medicine, No. 16, Airport Road, Baiyun District, Guangzhou, 510405 Guangdong People’s Republic of China; 7 Department of Liver Surgery, The First Affiliated Hospital of Sun Yat-Sen University, No. 58, Zhong Shan Road 2, Yuexiu District, Guangzhou, 510080 Guangdong People’s Republic of China

**Keywords:** Hepatocellular carcinoma, Recurrence, Magnetic resonance imaging, Deep learning, Nomograms

## Abstract

**Objectives:**

The accurate prediction of post-hepatectomy early recurrence in patients with hepatocellular carcinoma (HCC) is crucial for decision-making regarding postoperative adjuvant treatment and monitoring. We aimed to explore the feasibility of deep learning (DL) features derived from gadoxetate disodium (Gd-EOB-DTPA) MRI, qualitative features, and clinical variables for predicting early recurrence.

**Methods:**

In this bicentric study, 285 patients with HCC who underwent Gd-EOB-DTPA MRI before resection were divided into training (*n* = 195) and validation (*n* = 90) sets. DL features were extracted from contrast-enhanced MRI images using VGGNet-19. Three feature selection methods and five classification methods were combined for DL signature construction. Subsequently, an mp-MR DL signature fused with multiphase DL signatures of contrast-enhanced images was constructed. Univariate and multivariate logistic regression analyses were used to identify early recurrence risk factors including mp-MR DL signature, microvascular invasion (MVI), and tumor number. A DL nomogram was built by incorporating deep features and significant clinical variables to achieve early recurrence prediction.

**Results:**

MVI (*p* = 0.039), tumor number (*p* = 0.001), and mp-MR DL signature (*p* < 0.001) were independent risk factors for early recurrence. The DL nomogram outperformed the clinical nomogram in the training set (AUC: 0.949 vs. 0.751; *p* < 0.001) and validation set (AUC: 0.909 vs. 0.715; *p* = 0.002). Excellent DL nomogram calibration was achieved in both training and validation sets. Decision curve analysis confirmed the clinical usefulness of DL nomogram.

**Conclusion:**

The proposed DL nomogram was superior to the clinical nomogram in predicting early recurrence for HCC patients after hepatectomy.

**Key Points:**

*• Deep learning signature based on Gd-EOB-DTPA MRI was the predominant independent predictor of early recurrence for hepatocellular carcinoma (HCC) after hepatectomy.*

*• Deep learning nomogram based on clinical factors and Gd-EOB-DTPA MRI features is promising for predicting early recurrence of HCC.*

*• Deep learning nomogram outperformed the conventional clinical nomogram in predicting early recurrence.*

**Supplementary Information:**

The online version contains supplementary material available at 10.1007/s00330-023-09419-0.

## Introduction

Hepatic resection is the first-line treatment for patients with early-stage HCC and well-preserved liver function [1, 2]. However, HCC recurrence rate reaches 70% 5 years after surgery [3]. More than 80% of recurrences are intrahepatic, including intrahepatic metastases from a primary tumor (considered true recurrence) and de novo multicentric metastasis [4]. The poor prognosis of patients is related to intrahepatic metastases and mainly presents as early recurrence (within 2 years), whereas late recurrence (> 2 years) is more likely associated with underlying liver diseases, such as cirrhosis [5, 6]. Thus, early recurrence risk assessment in patients with HCC is clinically relevant.

Numerous tumor factors have been identified as predictors of early recurrence, such as microvascular invasion (MVI), surgical margin, tumor size, high lobular hepatitis activity, and poor Edmondson-Steiner grade [7–10]. However, most of the risk factors can only be obtained by postoperative pathology, and thus cannot be used to assess prognosis and develop treatment plans before hepatectomy. Medical imaging is a routine preoperative examination for patients with HCC. Previous studies have shown that quantitative parameters measured from medical imaging equipment, such as metabolic parameters, apparent diffusion coefficient values, and liver stiffness values from positron emission tomography/computed tomography [11], diffusion-weighted imaging [12], and magnetic resonance elastography [13], respectively, showed great efficiency and high clinical practicability in predicting the early recurrence of HCC after hepatectomy. Although magnetic resonance elastography is accurate in detecting liver fibrosis, the widespread application of this imaging modality in patients with HCC is limited due to its low specificity and its dependence on quantitative parameters; however, the cutoff of parameters was lack of criterion. Gadoxetate disodium (Gd-EOB-DTPA), a hepatobiliary-specific contrast agent that is widely used in patients with HCC, can better capture the perfusion and functional alterations, and hence may be more sensitive and accurate in HCC detection. Previous studies [14, 15] have also revealed that Gd-EOB-DTPA MRI is better than multidetector computed tomography (CT) and MRI using other contrast agents.

Several imaging features observed on Gd-EOB-DTPA MRI, including rim enhancement, arterial peritumoral enhancement, non-smooth tumor margin, satellite nodule, and peritumoral hypointensity on hepatobiliary phase (HBP) images [16, 17], are associated with early recurrence in patients with HCC; nevertheless, early recurrence prediction using MR features may be subjective and dependent on radiologist experience. Qualitative MR features are limited by image grayscale recognition, and therefore, much information related to tumor heterogeneity is lost.

Radiomics, involving the high-throughput extraction and mining of quantitative imaging features, is thought to capture the histological heterogeneity inherent to solid tumors [18]. In contrast to invasive tissue biomarkers, quantitative features retrieved from CT or MR images have demonstrated improved diagnostic and prognostic precision in patients with HCC, such as the preoperative prediction of MVI and recurrence-free survival [19]. However, more recent studies have shifted towards the field of deep learning (DL).

DL, a subset of machine learning, is a new diagnostic technology for mining internal information from medical images. DL can be applied to tumor segmentation [20], prognosis prediction [21, 22], and treatment response evaluation [23] by automatically extracting deep-learned or high-order image features. Among them, convolutional neural network (CNN) is famous for handling image classification tasks [24]; the three major operations of CNNs are convolution, activation, and pooling, and the entire process can be divided into two steps: the forward computation and the back propagation [25]. Additionally, DL has provided a preoperative prediction tool to guide postoperative clinical decision‐making regarding patients with HCC, such as HCC recurrence prediction after liver transplantation [26] and prognostic factor exploration in HCC pathological images [27]. Thus, the direct use of DL-based image features to predict prognosis would provide a promising non-invasive method to better individualize patient treatment.

Therefore, we aimed to investigate the feasibility of deep features extracted from Gd-EOB-DTPA MR images for predicting the early recurrence of HCC after curative resection. Furthermore, we evaluated the predictive performance of the DL-based nomogram incorporating deep features and significant clinical factors.

## Materials and methods

### Patients and dataset

**Fig. 1:**
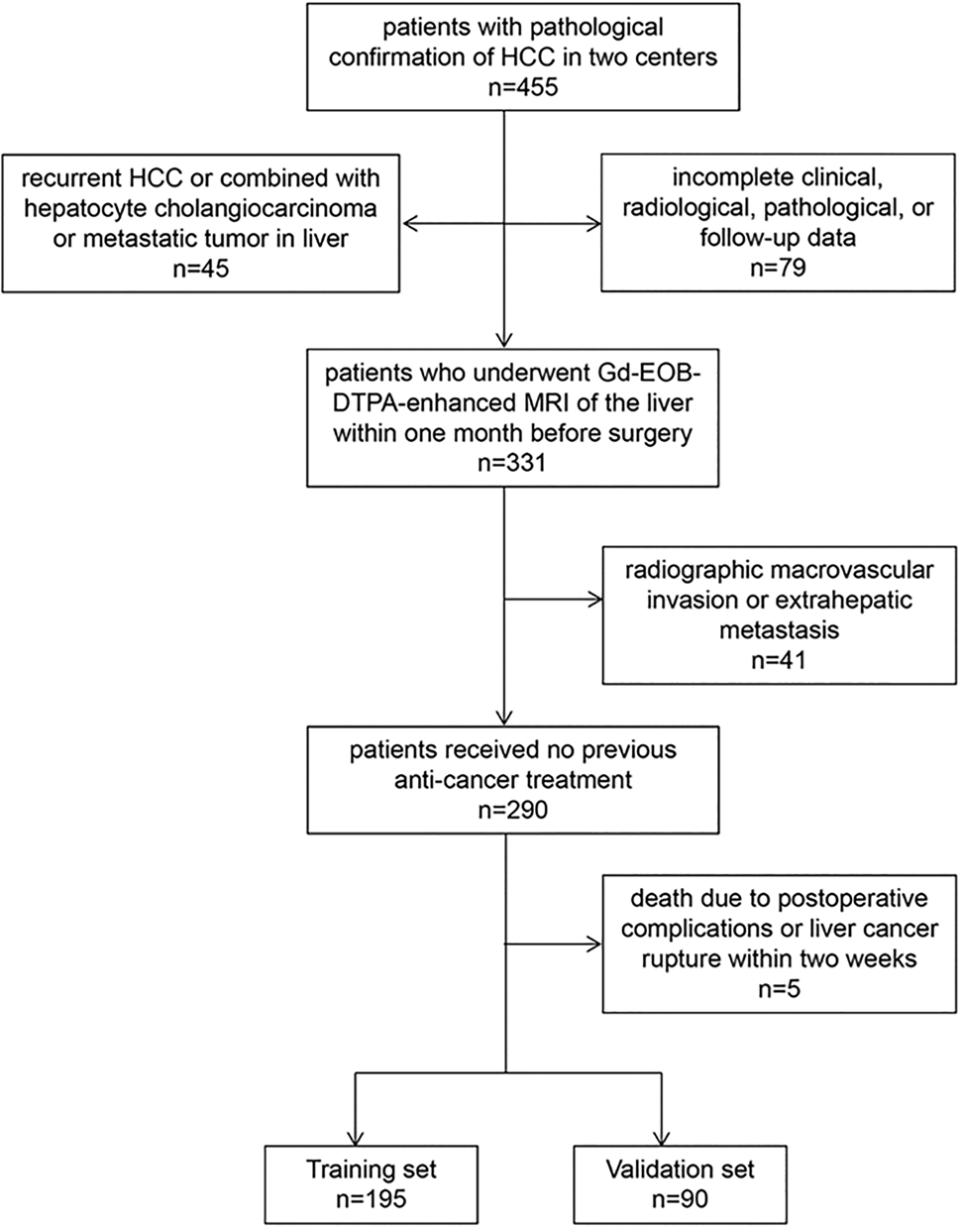
Flowchart of patient inclusion/exclusion for the two centers. Abbreviations: Gd-EOB-DTPA, gadoxetate disodium

Ethical approval was obtained for this retrospective study, and the requirement for informed consent was waived. Patients with suspected HCC who underwent Gd-EOB-DTPA MRI between January 2012 and September 2018 prior to curative resection were consecutively included. This study was performed at two centers: Sun Yat-Sen University Cancer Center (center 1) and Southern Medical University affiliated Zhujiang Hospital (center 2). The inclusion criteria were as follows: patients (a) with pathological confirmation of HCC; (b) with Barcelona Clinic Liver Cancer stage 0, A, or B HCC; (c) who received no previous anti-cancer treatment; and (d) who underwent Gd-EOB-DTPA MRI of the liver within 1 month before surgery. The exclusion criteria were as follows: patients (a) with recurrent HCC or combined with hepatocyte cholangiocarcinoma or metastatic tumor in the liver; (b) with radiographic macrovascular invasion or extrahepatic metastasis; (c) with incomplete clinical, radiological, pathological, or follow-up data; and (d) who died due to postoperative complications or liver cancer rupture within 2 weeks (Fig. 1). All patients including 227 patients in center 1 and 58 patients in center 2 were randomly divided into training and validation sets at in a 7:3 ratio.

Baseline clinicopathological data were collected from electronic medical records. Clinical data included demographics, time to early recurrence, and Barcelona Clinic Liver Cancer stage. Laboratory features included neutrophil count as well as hepatitis B virus DNA, α-fetoprotein (AFP), alanine aminotransferase, aspartate aminotransferase (AST), and γ-glutamyl transpeptidase levels. Pathologic data included MVI, defined as tumor emboli in a vascular space lined by endothelial cells on microscopy [28], tumor number, and histologic grade, which was evaluated as well differentiated, moderately differentiated, or poorly differentiated.

### Follow-up surveillance and clinical endpoint

All patients were followed up for at least 2 years after curative resection. Patients were screened for tumor recurrence through serum AFP level evaluation, ultrasonography, contrast-enhanced CT, or MRI of the chest and abdomen in the first month after surgery, once every 3 months thereafter during the first year, and every 6 months thereafter. The censored follow-up date was October 1, 2020.

The study endpoint was early recurrence, which was defined as one or more of the following events occurring within 2 years after curative resection: (a) presence of new hepatic lesions with typical imaging findings of HCC; (b) atypical imaging findings with biopsy or re-postoperative pathology-confirmed HCC, or postoperative transarterial chemoembolization (TACE) revealed tumor staining; and (c) extrahepatic metastases confirmed by typical imaging features or histological analysis.

### MRI acquisition and preprocessing

MR images of the arterial phase (AP), portal venous phase (PVP), and HBP were collected in this study. The MRI machines and parameters provided in sequences and parameters are listed in Supplementary Methods and Supplementary Table 1. Before the quantitative analysis, we preprocessed the MR images considering the influence of different MRI scanning parameters, protocols, and individual differences. First, an offset field correction by N4 algorithm in 3D Slicer software was applied to correct the inhomogeneity in gray level. Then, we mapped the gray values to a range of 0–255, to reduce the influence of different gray scales on gray quantization.

### Qualitative analysis of MR images

The qualitative analysis of MR image features was independently performed by three abdominal radiologists (M.Y., B.Z., and Z.J.G. with 5, 10, and 16 years of abdominal diagnosis experience, respectively). The radiologists were blinded to the radiological and pathologic reports. Reader 1 (M.Y.) observed the features twice, and the second observation was performed 2 weeks after the first observation.

**Fig. 2:**
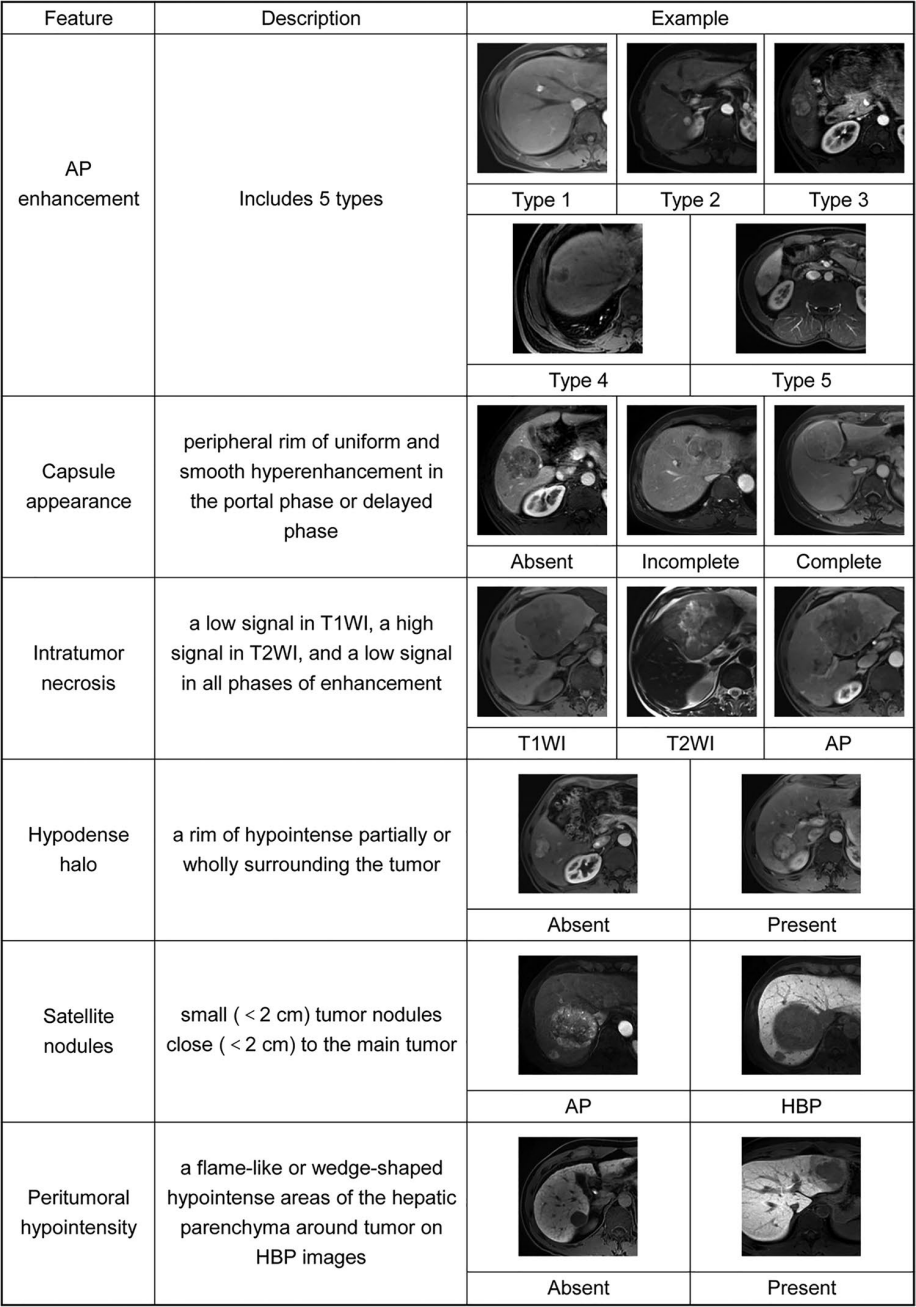
Definitions of features and representative MR images. Abbreviations: AP, arterial phase; Type 1, a homogeneous enhancement pattern with no increased arterial blood flow; Type 2, a homogeneous enhancement with increased arterial blood flow; Type 3, a heterogeneous enhancement included non-enhanced areas; Type 4, a heterogeneous enhancement pattern with irregular ring-like structures; Type 5, a heterogeneous and hypointense enhancement pattern; HBP, hepatobiliary phase

The MR features included the following: (a) tumor size, defined as the maximum diameter on transverse HBP images; (b) AP enhancement type (types 1 [homogeneous enhancement pattern with no increased arterial blood flow], 2 [homogeneous enhancement with increased arterial blood flow], 3 [heterogeneous enhancement containing non-enhanced areas], 4 [heterogeneous enhancement pattern with irregular ring-like structures] [29–31], and 5 [heterogeneous and hypointense enhancement pattern]); (c) capsule appearance (peripheral rim of uniform and smooth hyperenhancement in the portal or delayed phase, which is categorized into three groups [absent, incomplete, and complete]) [32]; (d) hypodense halo (a rim of hypointensity partially or wholly surrounding the tumor); (e) intratumor necrosis (a low signal on T1-weighted imaging, a high signal on T2-weighted imaging, and a low signal on all enhanced phases); (f) satellite nodules, defined as small (< 2 cm) tumor nodules close (< 2 cm) to the main tumor [33]; (g) peritumoral hypointensity, defined as flame-like or wedge-shaped hypointense areas of the hepatic parenchyma around the tumor on HBP images [34]. Figure 2 shows the MR features.

### Image segmentation and DL feature extraction

**Fig. 3:**
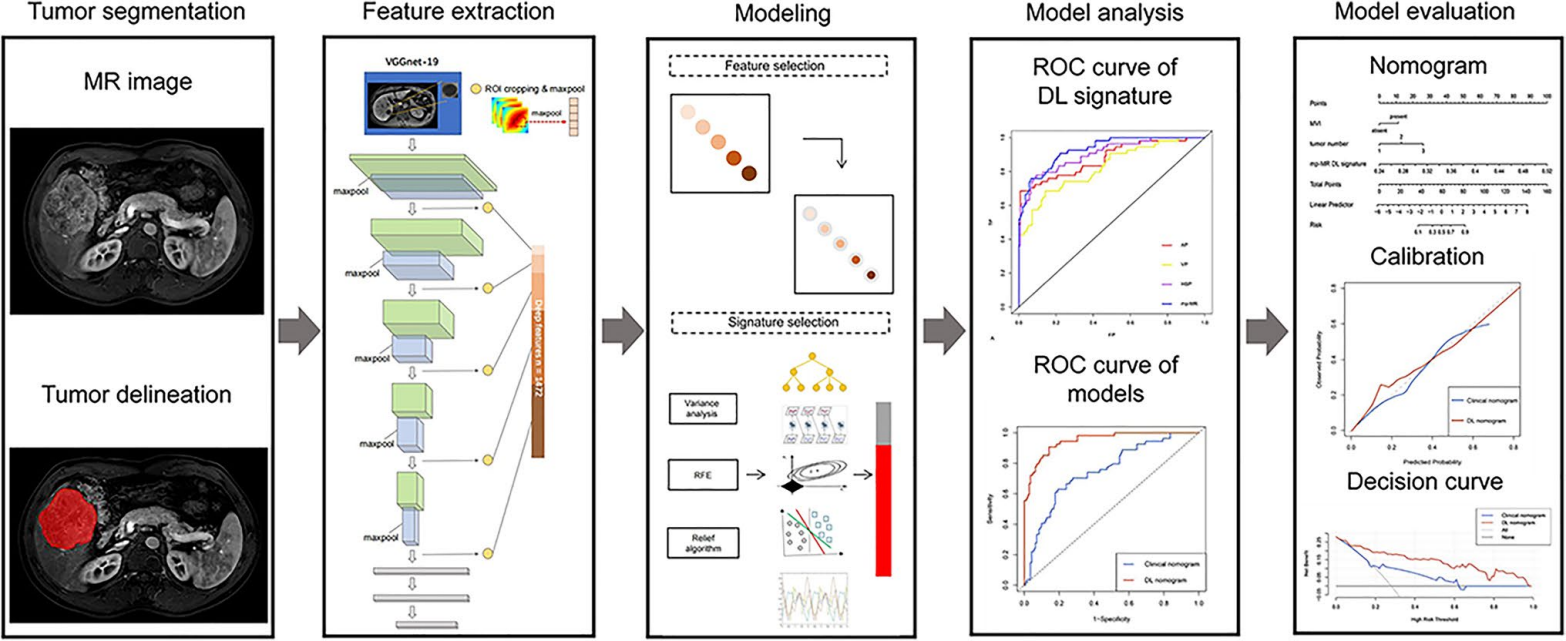
The workflow of deep learning analysis. Abbreviations: RFE, recursive feature elimination; DL, deep learning

The regions of interest were delineated around the tumor boundary on the largest dimension. A state-of-the-art architecture VGGNet-19 was then applied to extract 1472 DL features from the AP, PVP, and HBP images. The DL network contains five convolutional layers, four max-pooling layers, three fully connected layers, and a softmax layer. The DL workflow is shown in Fig. 3, and more details are provided in Supplementary Methods.

### Feature selection and DL signature development

To select the features strongly related to early recurrence, the DL features were subjected to the following steps: feature value preconditioning, de-redundancy, and dimensionality reduction; thereafter, machine learning methods were used to predict the status of outcome events and establish a DL signature that can predict early recurrence. All features were first normalized to the range of [0,1] by the minimum–maximum normalization method. Moreover, Spearman correlation analysis was added to retain DL features associated with the early recurrence of HCC (*p* < 0.05). Subsequently, the Pearson correlation coefficient (*r*) was used to remove one redundant feature with a lower *r* from the feature pairs (*r* > 0.9). The highly predictive features obtained were further screened by variance analysis, recursive feature elimination (RFE), and Relief algorithm. Five types of classifiers, namely random forest (RF), support vector machine, least absolute shrinkage and selection operator logistic regression (LASSO), AdaBoost, and Gaussian process (GP), were compared to identify the outcome status of early recurrence for every phase of the DL features.

### Clinical and DL analysis

Univariate logistic regression analysis was performed in the training set, and significant variables (*p* < 0.05) were entered into the multivariate logistic regression using the forward likelihood ratio method to identify the independent risk factors for early recurrence. A two-sided *p* < 0.05 was considered statistically significant. The nomogram was plotted based on multivariate logistic regression analysis findings.

Collinearity analysis of conventional clinical factors and DL signatures was also performed. The evaluation factors were tolerance and variance inflation factor (VIF); a tolerance value < 0.1 or a VIF value > 5 was considered to indicate collinearity between two variables.

We calculated the radiomics quality score (https://www.radiomics.world/rqs2) to assess the methodology, analysis, and reporting of our DL study [35].

### Statistical analysis

Comparisons between the training and validation sets were conducted using the chi-square test or Fisher’s exact test for categorical variables, whereas the Mann–Whitney *U* test was used for continuous variables. Intra-reader agreements of the qualitative MR imaging features were assessed using Cohen’s *κ* coefficient, and the inter-reader agreement was assessed by Fleiss’ *κ* statistics across three readers. Kappa (*κ*) statistics were qualitatively stratified by *κ* = 0.00–0.20, poor agreement; *κ* = 0.21–0.40, fair agreement; *κ* = 0.41–0.60, moderate agreement; *κ* = 0.61–0.80, good agreement; and *κ* = 0.81–1.00, excellent agreement [35].

The receiver operating characteristic curve analysis was employed to calculate the area under the curve (AUC), accuracy, sensitivity, and specificity. Comparisons between different DL signatures and models were implemented using the DeLong test. Model fit was assessed via calibration plots using 1000 bootstrap resamples. The clinical utility of the models was evaluated using decision curve analysis. Software and packages for statistical analyses are provided in Supplementary Methods**.** All statistical tests were two-sided, and a *p* < 0.05 was considered statistically significant.

## Results

### Clinical characteristics

We included 285 patients (men, *n* = 254; median age, 54.0 years; range, 13–79 years) whose data were divided into the training set (*n* = 195) and validation set (*n* = 90). Early recurrence occurred in 77 (27.0%) patients, and there was no difference in the early recurrence rate between the training set (27.7%, 54/195) and validation set (25.6%, 23/90). A total of 364 tumors were found in all patients, including 1, 2, 3, and 4 tumors in 223, 47, 13, and 2 patients, respectively. The clinical characteristics of the two groups are shown in Supplementary Table 2. No statistically significant difference was observed in baseline clinicopathological variables, pathologic variables, and qualitative MR features between the two sets (*p* = 0.113–1.000) except for the factor of etiology (*p* = 0.012).

Among all HCC patients in the early recurrence group, there were 52 (67.5%) patients with intrahepatic recurrence, including 48 (62.3%), 3 (3.9%), and 1 (1.3%) patients with typical CT/MR finding, re-postoperatively confirmed HCC, and TACE-revealed tumor staining, respectively; and 30 (39%) patients with extrahepatic recurrence, including 25 (32.5%) and 4 (5.2%) with typical CT/MR finding and histological analysis, respectively. Additionally, 5 (6.5%) patients had concurrent intra- and extrahepatic recurrences. The median time to early recurrence was 8 months (range, 1–24 months). The recurrence details are provided in Supplementary Table 6.

### Intra- and inter-reader agreement for qualitative MR images

Supplementary Table 5 presents the percentages of all features identified by the three readers as well as their intra- and inter-reader agreements and *κ* statistics for each imaging. Each MR feature showed an almost perfect intra-reader agreement (*κ* = 0.81–1.00). Regarding the inter-reader agreement among the three readers, the hypodense halo, peritumoral hypointensity, satellite nodules, capsule appearance, and intratumor necrosis showed good agreement (*κ* = 0.72–0.79); AP enhancement type and tumor size showed excellent agreement (*κ* = 0.84 and 0.97, respectively).

### DL signature development and validation

**Fig. 4:**
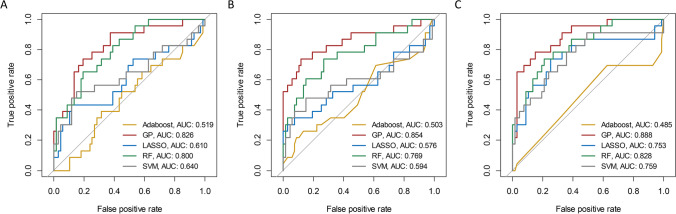
Performance comparisons among the five classifiers for the validation set: **A** AP images; **B** PVP images; **C** HBP images. Abbreviations: AP, arterial phase; PVP, portal venous phase; HBP, hepatobiliary phase; GP, Gaussian process; LASSO, least absolute shrinkage and selection operator logistic regression; RF, random forest; SVM, support vector machine

For identifying the outcome status of early recurrence in every phase of AP, PVP, and HBP features, the performance comparisons among five classifiers are shown in Fig. 4. It manifested that the GP classifier achieved the best performance for all the sequences with the AUCs of 0.826 (AP), 0.854 (PVP), and 0.888 (HBP), while the AdaBoost classifier showed almost no discrimination ability with the AUCs of 0.519 (AP), 0.503 (PVP), and 0.485 (HBP). In total, 99 DL features were selected from AP images using RFE, 93 DL features were screened from PVP images using Relief, and 99 DL features were identified from HBP images using Relief. All the optimal single-layered DL signatures were constructed using GP classifiers. All DL signatures showed significant differences between the two groups (all *p* < 0.05) in the training set (Table 1). Collinearity analysis was performed for variables with *p* < 0.05 after univariate analysis. The tolerance range of each variable was 0.750–0.970, and the VIF range was 1.031–1.333, indicating no collinearity between the variables (Supplementary Table 3).

**Table 1:** Difference analysis and univariate analysis of DL signature between groups with and without the early recurrence in the training set

	Phase	Early recurrence (*n* = 54)	Without early recurrence (*n* = 141)	Difference analysis	Univariate analysis	
				*p* value	OR (95%CI)	*p* value
DL signature	AP	0.450 (0.379, 0.545)	0.297 (0.266, 0.356)	< 0.001^*^	6.08 × 10^9^ (1.389 × 10^7^–9.627 × 10^12^)	< 0.001^*^
	PVP	0.390 (0.294, 0.507)	0.270 (0.245, 0.321)	< 0.001^*^	4.68 × 10^7^ (3.036 × 10^5^–1.781 × 10^10^)	< 0.001^*^
	HBP	0.434 (0.371, 0.515)	0.304 (0.272, 0.339)	< 0.001^*^	1.791 × 10^12^ (9.725 × 10^8^–1.753 × 10^16^)	< 0.001^*^
	mp-MR	0.425 (0.374, 0.498)	0.291 (0.273, 0.322)	< 0.001^*^	1.605 × 10^14^ (3.544 × 10^10^–4.259 × 10^18^)	< 0.001^*^

*OR*, odds ratio; *CI*, confidence interval; *DL*, deep learning; *AP*, arterial phase; *PVP*, portal venous phase; *HBP*, hepatobiliary phase; *mp-MR*, multiple sequences magnetic resonance. p^*^ < 0.05 indicates a significant difference

The AP DL signature achieved an AUC of 0.882 (95% confidence interval [CI]: 0.823–0.941) in the training set and 0.826 (95% CI: 0.755–0.897) in the validation set (Table 2); the PVP DL signature yielded an AUC of 0.822 (95% CI: 0.753–0.891) in the training set and 0.854 (95% CI: 0.780–0.928) in the validation set (Table 2), whereas the HBP-DL signature demonstrated an AUC of 0.909 (95% CI: 0.860–0.958) in the training set and 0.888 (95% CI: 0.833–0.943) in the validation set (Table 2). The multi-sequence MR (mp-MR) DL signature performance improved in both training and validation sets, with AUCs of 0.929 (95% CI: 0.829–0.966) and 0.894 (95% CI: 0.811–0.977) in the training and validation sets, respectively (Table 2). The DeLong test demonstrated that the mp-MR DL signature performance was better than that of the AP DL signature in both training and validation sets (*p* < 0.05) (Table 2).

**Table 2:** Predictive performance of different DL signatures in training and validation sets

	Datasets	Phase	Sensitivity (%)	Specificity (%)	Accuracy (%)	AUC (95%CI)	*p* value
DL signature	Training set	AP	76.4	84.3	82.1	0.882 (0.823–0.941)	0.048^*^
		PVP	67.3	85.0	80.0	0.822 (0.753–0.891)	0.001^*^
		HBP	78.2	90.7	87.2	0.909 (0.860–0.958)	0.115
		mp-MR	76.4	93.6	88.7	0.929 (0.829–0.966)	Ref
	Validation set	AP	73.9	79.1	77.8	0.826 (0.755–0.897)	0.019^*^
		PVP	73.9	86.6	83.3	0.854 (0.780–0.928)	0.259
		HBP	78.3	77.6	77.8	0.888 (0.833–0.943)	0.743
		mp-MR	78.3	89.6	86.7	0.894 (0.811–0.977)	Ref

*AUC*, area under the curve; *CI*, confidence interval; *AP*, arterial phase; *DL*, deep learning; *PVP*, portal venous phase; *HBP*, hepatobiliary phase; *mp-MR*, multiple sequences magnetic resonance. p^*^ < 0.05 indicates a significant difference

### Establishment of clinical and DL nomograms

**Fig. 5:**
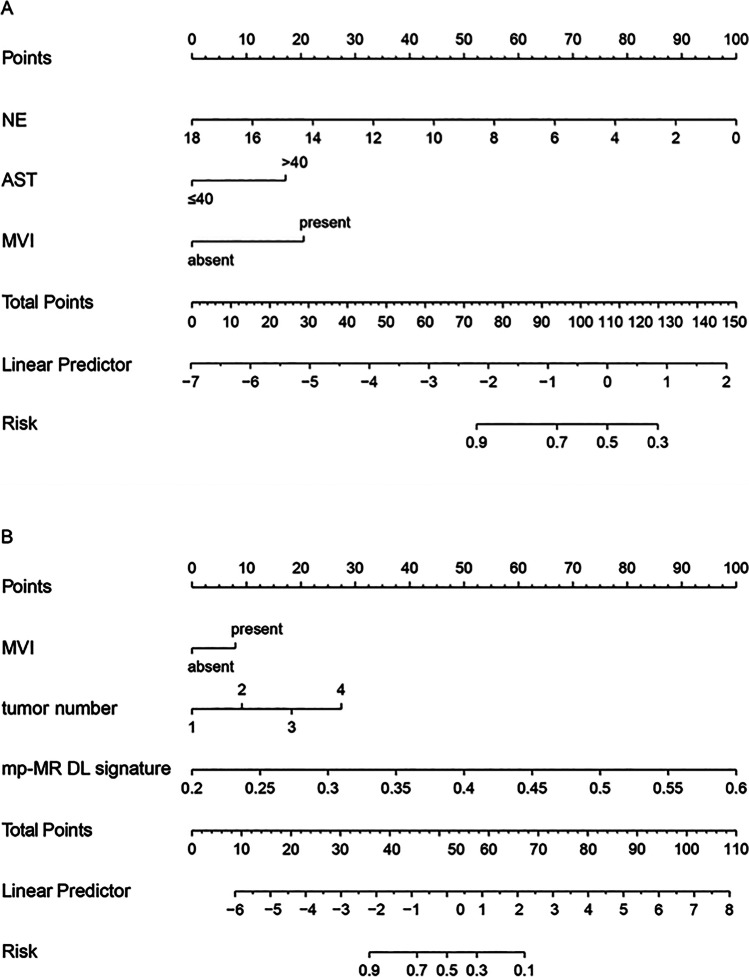
Clinical and deep learning nomograms. Clinical nomogram (**A**) was developed with NE, AST level, and MVI. Deep learning nomogram (**B**) was developed with MVI, tumor number, and mp-MR DL signature. Abbreviations: NE, neutrophil count. AST, aspartate aminotransferase. MVI, microvascular invasion; mp-MR, multiple sequences magnetic resonance; DL, deep learning

**Fig. 6:**
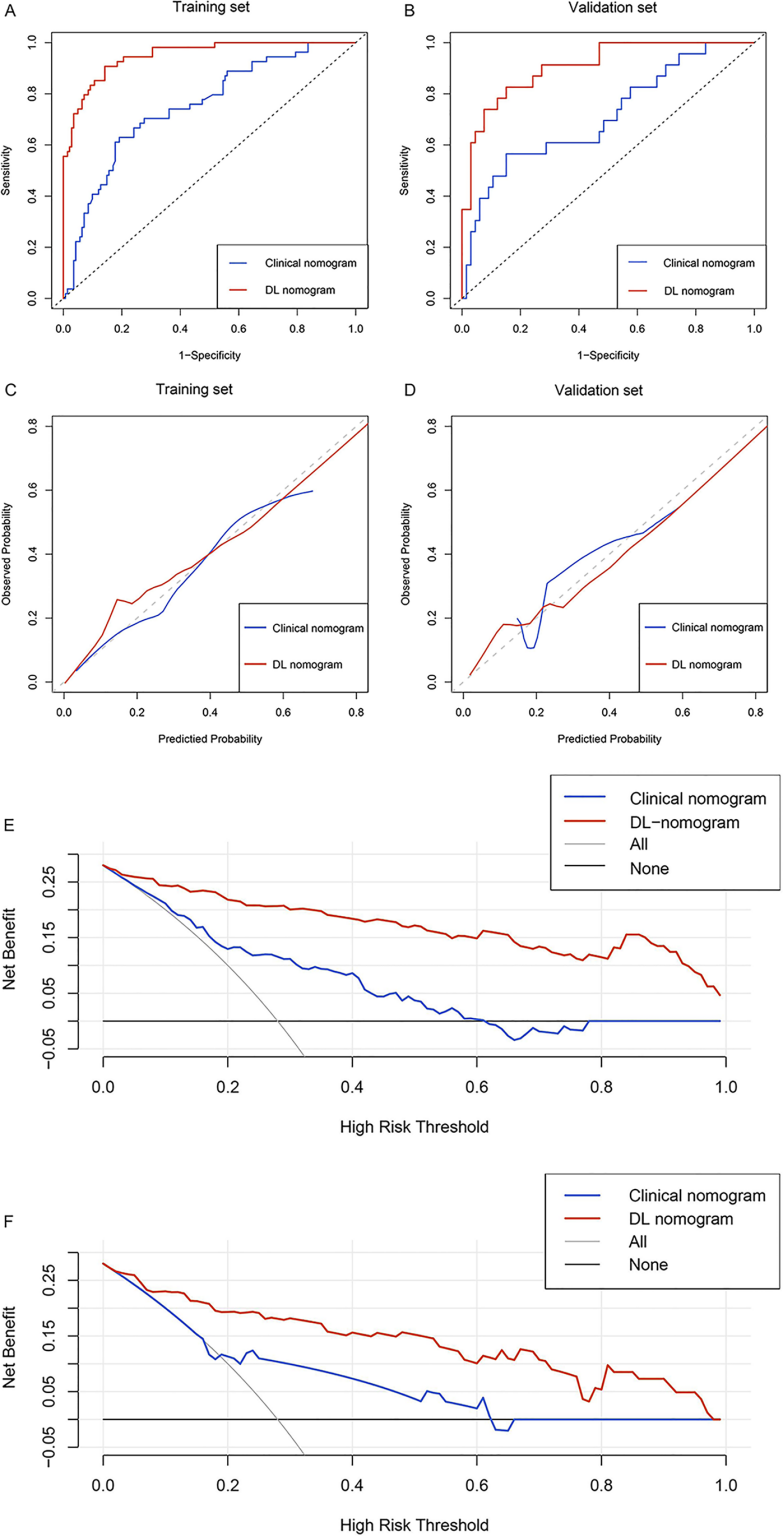
Discrimination, calibration, and clinical usefulness of the nomograms. The receiver operating characteristic curves for clinical nomogram and deep learning nomogram in the training (**A**) and validation (**B**) sets. Calibration curves for the clinical and deep learning nomograms in the training (**C**) and validation (**D**) sets. The *y*-axis represents the actual early recurrence rate, the *x*-axis represents the predicted early recurrence, and the diagonal dashed line represents the ideal prediction by a perfect model. Decision curve analysis for the clinical and deep learning nomograms in the training (**E**) and validation (**F**) sets. The *y*-axis represents the net benefits, and the *x*-axis represents the threshold probability. The deep learning nomogram (red line) had a good net benefit compared with the clinical nomogram (blue line), simple strategies such as follow-up of all patients (gray line), or no patients (horizontal black line) across the majority range of threshold probabilities. Abbreviation: DL, deep learning

After univariate analysis, six clinical factors, namely neutrophil count, AST level, γ-glutamyl transpeptidase level, MVI, tumor number, and histologic grade (*p* < 0.05), were candidate factors for multivariate analysis (Supplementary Table 4); however, only neutrophil count, AST level, and MVI were identified as independent risk factors for early recurrence of HCC. The clinical nomogram was constructed based on the three factors (Table 3; Fig. 5A) which achieved an AUC of 0.751 (95% CI: 0.674–0.827) in the training set and 0.715 (95% CI: 0.586–0.843) in the validation set (Table 4; Fig. 6A, B).

**Table 3:** Multivariate regression analysis of the groups with and without early recurrence in the training set

Variables	Clinical nomogram		DL nomogram	
	Odd ratios (95%CI)	*p* value	Odd ratios (95%CI)	*p* value
NE	0.70 (0.52–0.93)	0.014^*^	NA	NA
AST	3.07 (1.52–6.22)	0.002^*^	NA	NA
MVI	3.82 (1.87–7.80)	< 0.001^*^	3.06 (1.06–8.82)	0.039^*^
Tumor number	NA	NA	3.61 (1.64–7.92)	0.001^*^
mp-MR DL signature	NA	NA	1.61 × 10^15^ (5.63 × 10^10^–4.63 × 10^19^)	< 0.001^*^

*DL*, deep learning; *NE*, neutrophil count; *NA*, not applicable; *AST*, aspartate aminotransferase; *MVI*, microvascular invasion; *mp-MR*, multiple sequences magnetic resonance. p^*^ < 0.05 indicates a significant difference

**Table 4:** Predictive performances of nomograms on the training and validation sets

Model	Training set				Validation set			
	AUC (95%CI)	Sensitivity (%)	Specificity (%)	*p* value	AUC (95%CI)	Sensitivity (%)	Specificity (%)	*p* value
Clinical nomogram	0.751 (0.674–0.827)	63.0	80.9	< 0.001*	0.715 (0.586–0.843)	56.5	85.1	0.002*
mp-MR DL signature	0.929 (0.829–0.966)	76.4	93.6	0.099	0.894 (0.811–0.977)	78.3	89.6	0.378
DL nomogram	0.949 (0.919–0.980)	90.7	85.5	Ref	0.909 (0.842–0.976)	82.6	85.1	Ref

*AUC*, area under the curve; *CI*, confidence interval; *mp-MR*, multiple sequences magnetic resonance; *DL*, deep learning. p^*^ < 0.05 indicates a significant difference

Thereafter, the above-mentioned five clinical factors and the mp-MR DL signature were included in the multivariate analysis, and tumor number, MVI, and mp-MR DL signature were identified as independent risk factors for early recurrence (*p* < 0.05). We developed a DL nomogram incorporating the tumor number, MVI, and mp-MR DL signature (Table 3; Fig. 5B), which significantly outperformed the clinical nomogram, yielding an AUC of 0.949 (95% CI: 0.919–0.980) in the training set and 0.909 (95% CI: 0.842–0.976) in the validation set (Table 4; Fig. 6A, B). The DeLong test showed a significant difference between the clinical and DL nomograms in the training set (*p* < 0.001) and validation set (*p* = 0.002) (Table 4). Figure 6C and D demonstrate good DL nomogram calibration. The decision curve showed that the DL nomogram had a higher net benefit than the clinical nomogram (Fig. 6E, F). This DL study scored 40 points (60.6%) (Supplementary Material_RQS).

## Discussion

We used the DL approach to explore the informative features from Gd-EOB-DTPA MRI images that were associated with early recurrence of HCC and established three single-layered DL signatures and an mp-MR DL signature fused with three-phase MR sequences. The results showed that the mp-MR DL signature was better than the three single-layered DL signatures. Subsequently, the DL nomogram was constructed by integrating tumor number, MVI, and the mp-MR DL signature, which achieved higher predictive accuracy and better net benefit than the clinical nomogram. This study demonstrated the incremental value of the DL nomogram compared with the conventional clinical nomogram.

Previous studies also revealed that Gd-EOB-DTPA MRI had a significantly higher sensitivity and overall accuracy for HCC diagnosis, especially small lesions, than multiphasic CT without substantial loss of specificity [36, 37]. Hence, we chose Gd-EOB-DTPA MRI instead of CT images to predict the early recurrence in patients with HCC. As recent studies reported [38, 39], obtaining high stability of features from different MR scanners becomes an increasingly common challenge in radiomics or DL field. As the data in our study were obtained from different MR scanners, we did offset field correction and gray normalization to reduce the potential affect. The preprocessing procedure is usually conducted in other studies [10, 40, 41]. The challenge of eliminating feature variability may be addressed through optimization tools developed for DL, via standardization of imaging protocols [42], and through prospective studies. In addition, we demonstrated that the HBP signature yielded a higher AUC and sensitivity than AP signature and PVP signature. Well-defined tumor margins on HBP images allow for a more accurate tumor delineation than those on AP and PVP images, in which the tumor margins could be affected by peritumoral enhancement, capsule appearance, and a hypodense halo.

Some previous studies have explored the feasibility of predicting early postoperative HCC recurrence. An et al. [17] and Zhang et al. [44] developed nomograms containing clinic-radiological variables to predict the early recurrence in patients with HCC, achieving AUCs of 0.783–0.846 in the validation cohorts. Chan et al. [45] constructed a preoperative model (early recurrence after surgery for liver tumor [ERASL]-pre) and a postoperative model (ERASL-post) based on gender, AFP level, MVI, tumor size, and tumor number, with AUCs ranging from 0.614 to 0.736 for the ERASL-pre and 0.653 to 0.763 for the ERASL-post in the derivation cohorts from different countries or districts. In our study, we also built a clinical nomogram and the diagnostic efficacy was similar to those in previous studies, with AUCs of 0.751 and 0.715 in the training and validation sets, respectively. Although these data are commonly and easily available in clinical practice, the two major problems encountered were non-uniformity in the standardization of clinical factors and the inability to handle high-dimension features using traditional statistical methods [46].

In recent years, artificial intelligence has emerged as an effective tool to demonstrate multi-modal patient data [47]. Radiomics is a recently emerged technology that extracts a large number of quantitative image features from standard-of-care medical imaging using data-characterization algorithms. Zhang et al. [48] and Zhao et al. [40] constructed a nomogram by integrating radiomic score and clinic-radiological factors, with AUCs of 0.844 and 0.873, respectively. Kim et al. [49] developed a combined clinicopathologic-radiomic model via random survival forest algorithm, which acquired a *C*-index of 0.716. In the latest research, the DL methods have outperformed the radiomics features in many tasks including lesion detection [50], prognosis prediction [51], and multimodal image registration [52]. The image-based DL technology has been widely applied in the HCC field of mass differentiation, treatment response, and prognosis [10, 53–55]. Song et al. [56] established a DL model using CNN in eight MRI sequences to predict the presence of MVI, which acquired an AUC of 0.931. Zhang et al. [57] constructed an integrated nomogram combining clinical features and DL signatures based on contrast CT to improve overall survival prediction in HCC patients treated with TACE plus sorafenib, with a *C*-index of 0.730 in the validation set. Based on the successful applications of DL in patients with HCC, we explored the VGGnet-19, which generally performs a more robust and automatic image analysis without export’s intervention [58], to extend the associations between DL and prognosis of HCC. Additionally, we employed 2D regions of interest to establish the DL signatures, which showed great performance; this finding corroborates with previous studies [57, 59, 60], wherein AUCs of 0.826–0.894 were achieved in the validation set. It is probably because the DL signatures could make predictions by capturing both global and local features of tumors, and it comprehensively reflected on the tumor size and heterogeneity, which were established prognostic factors [61, 62]. Thus, it may also explain the lack of tumor size as an independent risk factor because the DL signatures have already contained the information of tumor size which belongs to the tumor global feature. Nevertheless, this is subject to our future research.

In the field of feature engineering, different machine learning–based dimensionality reduction techniques have distinct mathematical senses and inherent limitations; thus, multiple algorithms should be combined to select robust features [42, 63]. Previous studies also proved that the combination of different dimensional reduction methods with several machine learning methods could maximize model diagnostic performance. Dai et al. [64] reported that feature selection and modeling methods could potentially affect prediction models. The optimal radiomic model for MVI evaluation was constructed using a gradient boosting decision tree classifier, which outperformed logistic regression, support vector machine, and random forest. Using 21 combination methods (including three feature selection methods and seven classification methods), Ni et al. [65] identified LASSO plus gradient boosting decision tree as the optimal combination for predicting MVI in patient with HCC. In the present study, we compared three feature selection methods with five classification methods to determine the best combination and found that RFE or Relief combined with GP classifier achieved the optimal performance in building DL signatures. Consequently, it is necessary to implement performance comparisons of different machine learning methods, which was absent in previous deep learning studies for HCC [43, 56, 57].

Our study has several limitations. First, the retrospective nature of the study may induce inevitable selection bias. Second, although we collected patient data from two centers, we were unable to perform an external validation because the sample size of center 2 was small; thus, multicenter studies are needed to validate the generalization ability of the proposed DL nomogram. Third, our study did not explore the role of DL features extracted from non-contrast MR sequences such as T1-weighted imaging, T2-weighted imaging, and diffusion-weighted imaging for predicting prognosis. Finally, the value of the DL model for improving long-term survival in patients with HCC remains unclear, and the differences between DL model–assisted and non-assisted practices warrant further study to prove the clinical applicability of the DL model.

In conclusion, we developed a DL nomogram that incorporates clinical factors and Gd-EOB-DTPA MRI biomarkers; furthermore, the DL nomogram was more effective in early recurrence prediction and postoperative surveillance than the traditional clinical nomogram in patients with HCC following surgical resection.

## Supplementary Information

Below is the link to the electronic supplementary material.

Supplementary file1 (DOCX 185 KB)

## Data Availability

The data that support the findings of this study are available on reasonable request from the corresponding author. The data are not publicly available due to privacy or ethical restrictions.

## Abbreviations

AFP: α-fetoprotein
AP: Arterial phase
AST: Aspartate aminotransferase
AUC: Area under the curve
CNN: Convolutional neural network
DL: Deep learning
ERASL: Early recurrence after surgery for liver tumor
Gd-EOB-DTPA: Gadoxetate disodium
GP: Gaussian process
HBP: Hepatobiliary phase
HCC: Hepatocellular carcinoma
LASSO: Least absolute shrinkage and selection operator logistic regression
mp-MR: Multiple sequences magnetic resonance
MVI: Microvascular invasion
PVP: Portal venous phase
RFE: Recursive feature elimination
TACE: Transarterial chemoembolization

## Declarations

### Guarantor

The scientific guarantor of this publication is Xianyue Quan.

### Statistics and biometry

No complex statistical methods were necessary for this paper.

### Methodology

• Retrospective

• Diagnostic or prognostic study

• Multicenter study
