# Supplementary material for: Deep learning nomogram based on Gd-EOB-DTPA MRI for predicting early recurrence in hepatocellular carcinoma after hepatectomy
Source: Eur Radiol. 2023 Feb 14;33(7):4949–61. doi: 10.1007/s00330-023-09419-0 (PMC10289921; doi:10.1007/s00330-023-09419-0)
Supplement: Supplementary file 1 — Supplementary file1 (DOCX 185 KB) [file 330_2023_9419_MOESM1_ESM.docx]

**Supplementary Materials**

**Supplementary Figure 1.** **Performance comparisons of the DL nomogram for two centers enrolled in this study**


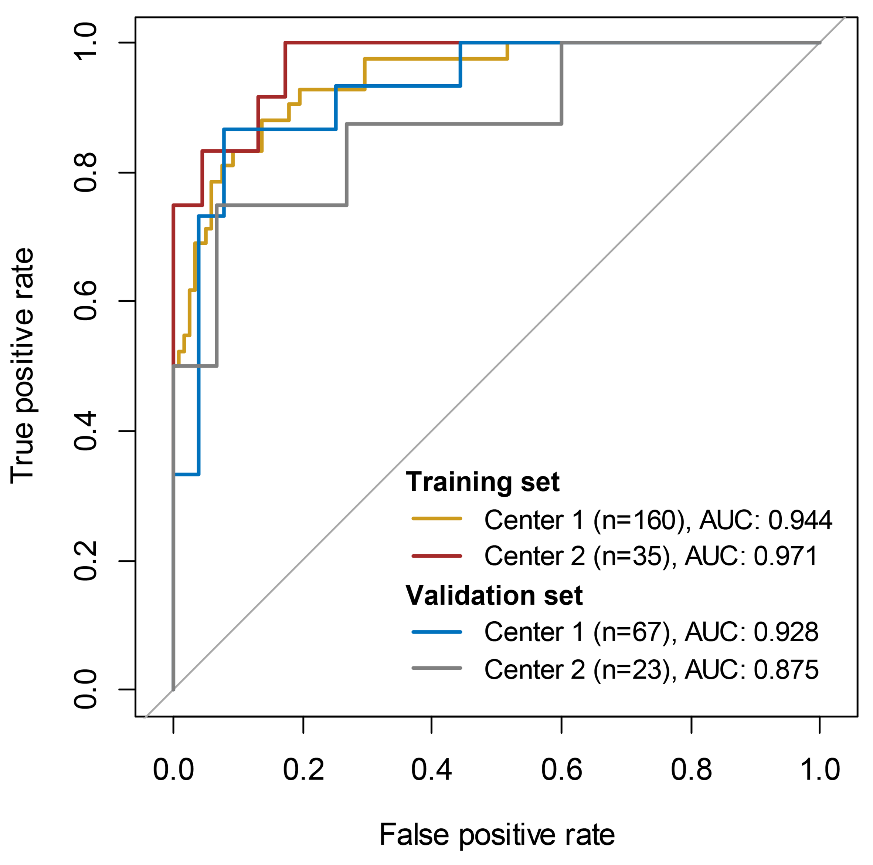


**Note:** Center 1, Sun Yat-Sen University Cancer Center. Center 2, Southern Medical University affiliated Zhujiang Hospital. AUC, Area under the curve.

**Supplementary Methods**

**MR imaging acquisition**

All patients were instructed to fast for 4-6 h before the MRI scan and received breath-hold training. The scanning range included the whole liver (from the diaphragmatic dome to the lower edge of the liver). In center 1, MRI scans of 94 patients were performed using Discovery MR 750 (3.0T, GE Healthcare, Milwaukee, Wisconsin, USA), 67 patients were examined using Achieva (3.0T, Philips Healthcare, Netherlands), and 66 patients were examined using Trio Tim (3.0T, Siemens Healthcare, Erlangen, Germany), with a 16-channel phased-array body coil. In center 2, MRI scans of 40 patients were performed using Ingenia (3.0 T, Philips Healthcare, Netherlands), 18 patients were examined using Achieva (3.0T, Philips Healthcare, Netherlands), and both scanners used a 16-channel phased-array body coil. After a scan of the unenhanced phase, MR images of the arterial phase (AP; 20-30 s), portal venous phase (PVP; 50-60 s), transitional phase (2-3 min), and hepatobiliary phase (HBP; 20 min) were obtained using a liver-specific contrast agent (Gd-EOB-DTPA; Primovist®, Bayer Schering Pharma AG, Berlin, Germany); the contrast agent was administered intravenously at a dose of 0.1 ml/kg at a rate of 1 ml/s and followed immediately by a 20 -ml saline (0.1 ml/kg) flush.

**Image segmentation and DL feature extraction**

All preoperative MR images were obtained from the picture archiving and communication system (PACS). The regions of interest of tumor were manually delineated by reader 1 (M.Y, a junior radiologist with 5 years of abdominal diagnosis experience) around the boundary of the largest dimension of the tumor on the transverse slice from the AP, PVP, and HBP MR images using the ‘Add tool’ in the ‘2D Tools’ option in the ‘Image Navigator’ toolbar of the MITK workbench (version 2018.4.0, <https://www.mitk.org>).

The segmentation of ROIs were as follows: first, DICOM images of the AP were imported into MITK workbench (version 2018.4.0, https://www.mitk.org), while images of the same case were imported into the software RadiAnt DICOM viewer (<https://www.radiantviewer.com/>) to assist in tumor observation; second, to clearly show the real tumor boundary, the optimal values of window width and window position were adjusted in the two software mentioned above before tumor delineation; third, since the sequences of T2WI, PVP, and HBP usually show the tumor boundary distinctively, as a reference, the same transverse images of T2WI, PVP, and HBP were placed adjacent to the AP images for tumor boundary identification; finally, the potential tumor region was properly enlarged using the MITK workbench, and the observed tumor boundary was manually delineated.

In this study, we used VGGNet-19 pretrained on the ImageNet dataset (www.image-net.org), but removed the fully connected subsequent layers, leaving only the convolution layers as the DL feature extractor and fixing its pre-trained weights. The processed network, referred to as the VGGNet-19 DL feature extractor. DL feature extraction was implemented using Keras with TensorFlow as the backend.

MR images were grayscale (single-channel images), which may have some limitations for feature extraction, while three-channel and 224 × 224 pixel RGB input images were required for the pretrained VGGNet-19. The largest slice of the tumor region in the MR images was duplicated into three channels (512 * 512 * 3) as the input of the VGGNet-19 DL feature extractor. The smallest rectangular region to fit the tumor of the representative slice was calculated and mapped to the feature maps using different convolution blocks to obtain the corresponding feature maps of interest. The regions were processed through global max pooling for each block to obtain one-dimensional feature vectors and finally connected to obtain all DL features from each MRI phase.

**Statistical analysis**

Chi-square test, Fisher’s exact test and Mann-Whitney U test were conducted using SPSS (version 26.0, IBM, Armonk, NY, USA)., DL feature extraction and modeling were performed using Python (version 3.7.6, https://www.python.org/) and Pycharm (version 2020.1.5, https://www.jetbrains.com /pycharm/). Intra- and inter-reader variability, model construction, evaluation, and comparison were conducted using R software (version 4.0.1, https://cran.r-project.org/bin/windows/ base/old/4.0.1). The R packages included “irr”, “rms”, “pROC”, “graphics”, “riskRegression”, and “rmda”.

**Supplementary Table 1. MR Imaging Acquisition**

| **MR scanners** | **Sequence** | **Parameters** | | | | | | |
| --- | --- | --- | --- | --- | --- | --- | --- | --- |
|  |  | **TR (ms)** | **TE (ms)** | **FOV (mm)** | **Matrix** | **Reverse angle (°)** | **Band width** | **Thickness (mm)** |
| **Institution 1** |  |  |  |  |  |  |  |  |
| Discovery MR 750, GE  Healthcare | T1WI | 4.6 | 1.7 | 300×400 | 288×208 | 15 | 558 | 4 |
|  | T2WI | 7058.8 | 104.2 | 300×400 | 320×320 | 142 | 326 | 5 |
|  | T1WI + C | 4.0 | 1.7 | 300×400 | 288×208 | 15 | 326 | 4 |
| Achieva TX,  Philips Healthcare | T1WI | 3.1 | 1.5 | 328×350 | 312×314 | 10 | 724 | 6 |
|  | T2WI | 1035.1 | 80 | 328×350 | 432×351 | 90 | 579 | 6 |
|  | T1WI + C | 3.1 | 1.5 | 328×350 | 312×314 | 10 | 724 | 6 |
| Achieva TX,  Philips Healthcare | T1WI | 0 | 0 | 328×350 | 236×234 | 10 | 1977 | 2.5 |
|  | T2WI | 776 | 80 | 328×350 | 268×217 | 90 | 518 | 5 |
|  | T1WI + C | 0 | 0 | 328×350 | 236×234 | 10 | 1977 | 2.5 |
| Trio Tim,  Siemens Healthcare | T1WI | 4.1 | 1.8 | 275×400 | 320×176 | 9 | 446 | 3 |
|  | T2WI | 5282 | 83 | 399×299 | 384×202 | 150 | 260 | 5 |
|  | T1WI + C | 4.2 | 1.8 | 275×400 | 320×176 | 9 | 446 | 3 |
| **Institution 2** |  |  |  |  |  |  |  |  |
| Ingenia,  Philips Healthcare | T1WI | 0 | 0 | 320×280 | 180×158 | 10 | 1977 | 3 |
|  | T2WI | 836 | 80 | 400×156 | 168×200 | 90 | 518 | 6 |
|  | T1WI + C | 0 | 0 | 294×280 | 156×161 | 10 | 1977 | 2.5 |

**Note:** +C, contrast-enhanced.

**Supplementary Table 2. Baseline characteristics of the training and validation sets**

| **Characteristics** | **Total**  **(n = 285)** | **Training set**  **(n = 195)** | **Validation set**  **(n = 90)** | ***P*-value** |
| --- | --- | --- | --- | --- |
| **Age (years)** | 54.0  (45.0-61.0) | 52.0  (45.0-60.0) | 56.5  (45.0-62.0) | 0.113 |
| **Gender** |  |  |  | 0.153 |
| Male | 254 (89.1) | 170 (87.2) | 84 (93.3) |  |
| Female | 31 (10.9) | 25 (12.8) | 6 (6.7) |  |
| **Etiology** |  |  |  | 0.012 |
| HBV | 241 (84.6) | 173 (88.7) | 68 (75.6) |  |
| HCV | 5 (1.8) | 2 (1.0) | 3 (3.3) |  |
| HBV and HCV | 1 (0.4) | 0 | 1 (1.1) |  |
| Others | 38 (13.3) | 20 (10.3) | 18 (20) |  |
| **NE (10^9^/L)** | 3.6 (2.8-4.6) | 3.6 (2.9-4.6) | 3.4 (2.8-4.7) | 0.622 |
| **HBV-DNA (IU/ml)** | 3.9×10^3^ (0.0-4.7×10^5^) | 3.1×10^3^ (0.0-3.7×10^5^) | 8.4×10^3^ (0.0-8.3×10^5^) | 0.608 |
| **AFP (ng/ml)** |  |  |  | 0.513 |
| ≤400 | 232 (81.4) | 161 (82.6) | 71 (78.9) |  |
| ＞400 | 53 (18.6) | 34 (17.4) | 19 (21.1) |  |
| **ALT (U/L)** |  |  |  | 0.670 |
| ≤50 | 206 (72.3) | 139 (71.3) | 67 (74.4) |  |
| ＞50 | 79 (27.7) | 56 (28.7) | 23 (25.6) |  |
| **AST (U/L)** |  |  |  | 0.484 |
| ≤40 | 204 (71.6) | 137 (70.3) | 67 (74.4) |  |
| ＞40 | 81 (28.4) | 58 (29.7) | 23 (25.6) |  |
| **GGT (U/L)** |  |  |  | 0.600 |
| ≤60 | 176 (61.8) | 118 (60.5) | 58 (64.4) |  |
| ＞60 | 109 (38.2) | 77 (39.5) | 32 (35.6) |  |
| **BCLC stage** |  |  |  | 0.336 |
| 0 | 61 (21.4) | 43 (22.1) | 18 (20) |  |
| A | 186 (65.3) | 130 (66.7) | 56 (62.2) |  |
| B | 38 (13.3) | 22 (11.3) | 16 (17.8) |  |
| **Tumor size (mm)** |  |  |  | 0.391 |
| ≤30 | 150 (52.6) | 106 (54.4) | 44 (48.9) |  |
| ＞30 | 135 (47.4) | 89 (45.6) | 46 (51.1) |  |
| **AP enhancement type** |  |  |  | 0.960 |
| Type 1 | 11 (3.9) | 8 (4.1) | 3 (3.3) |  |
| Type 2 | 62 (21.8) | 43 (22.1) | 19 (21.1) |  |
| Type 3 | 168 (58.9) | 112 (57.4) | 56 (62.2) |  |
| Type 4 | 20 (7.0) | 15 (7.7) | 5 (5.6) |  |
| Type 5 | 24 (8.4) | 17 (8.7) | 7 (7.8) |  |
| **Capsule appearance** |  |  |  | 1.000 |
| Absent | 101 (35.4) | 69 (35.4) | 32 (35.6) |  |
| Incomplete | 69 (24.2) | 47 (24.1) | 22 (24.4) |  |
| Complete | 115 (40.4) | 79 (40.5) | 36 (40) |  |
| **Hypodense halo** |  |  |  | 0.383 |
| Absent | 259 (90.9) | 175 (89.7) | 84 (93.3) |  |
| Present | 26 (9.1) | 20 (10.3) | 6 (6.7) |  |
| **Intratumor necrosis** |  |  |  | 0.402 |
| Absent | 201 (70.5) | 141 (72.3) | 60 (66.7) |  |
| Present | 84 (29.5) | 54 (27.7) | 30 (33.3) |  |
| **Satellite nodules** |  |  |  | 0.782 |
| Absent | 270 (94.7) | 184 (94.4) | 86 (95.6) |  |
| Present | 15 (5.3) | 11 (5.6) | 4 (4.4) |  |
| **Peritumoral hypointensity** |  |  |  | 1.000 |
| Absent | 257 (90.2) | 176 (90.3) | 81 (90) |  |
| Present | 28 (9.8) | 19 (9.7) | 9 (10) |  |
| **MVI** |  |  |  | 0.666 |
| Absent | 210 (73.7) | 142 (72.8) | 68 (75.6) |  |
| Present | 75 (26.3) | 53 (27.2) | 22 (24.4) |  |
| **Tumor number** |  |  |  | 0.478 |
| 1 | 223 (78.2) | 155 (79.5) | 68 (75.6) |  |
| 2 | 47 (16.5) | 30 (15.4) | 17 (18.9) |  |
| 3 | 13 (4.6) | 8 (4.1) | 5 (5.6) |  |
| 4 | 2 (0.7) | 2 (1.0) | 0 |  |
| **Histologic grade** |  |  |  | 0.251 |
| well differentiated | 34 (11.9) | 21 (10.8) | 13 (14.4) |  |
| moderately differentiated | 147 (51.6) | 107 (54.9) | 40 (44.4) |  |
| poorly differentiated | 104 (36.5) | 67 (34.4) | 37 (41.4) |  |

**Note:** Data are presented as number (%) or median (interquartile range, IQR). HBV, hepatitis B virus; HCV, hepatitis C virus; NE, neutrophil count; AFP, alpha-fetoprotein; ALT, alanine amino-transferase; AST, aspartate amino-transferase; GGT, γ-glutamyl transpeptadase; BCLC, barcelona clinic liver cancer; AP, arterial phase; Type 1, a homogeneous enhancement pattern with no increased arterial blood flow; Type 2, a homogeneous enhancement with increased arterial blood flow; Type 3, a heterogeneous enhancement included non-enhanced areas; Type 4, a heterogeneous enhancement pattern with irregular ring-like structures; Type 5, a heterogeneous and hypointense enhancement pattern; MVI, microvascular invasion.

**Supplementary Table 3. Collinearity analysis of clinical factors and mp-MR DL signature**

| **Variables** | Collinearity Statistics | |
| --- | --- | --- |
|  | Tolerance | VIF |
| NE | 0.970 | 1.031 |
| AST | 0.775 | 1.291 |
| GGT | 0.750 | 1.333 |
| MVI | 0.941 | 1.063 |
| Tumor number | 0.921 | 1.085 |
| mp-MR DL signature | 0.837 | 1.195 |

**Note:** VIF, variance inflation factors; NE, neutrophil count; AST, aspartate amino-transferase; GGT, γ-glutamyl transpeptidase; MVI, microvascular invasion; mp-MR, multiple sequences magnetic resonance; DL, deep learning.

**Supplementary Table 4. Univariate analysis of the groups with and without early recurrence in the training set**

| **Variables** | **Early recurrence**  **(n = 54)** |  | **Without early recurrence** |  | **Difference analysis** |  | **Univariate analysis** |  |
| --- | --- | --- | --- | --- | --- | --- | --- | --- |
|  |  |  | **(n = 141)** |  | ***P-value*** |  | **OR (95%CI)** | ***P-value*** |
| **Age (years)** | 51.50 (41.75-58.25) |  | 52.00 (46.00-60.50) |  | 0.241 |  |  | 0.123 |
| **Gender** |  |  |  |  | 0.812 |  |  | 0.659 |
| Male | 48 (89) |  | 122 (87) |  |  |  |  |  |
| Female | 6 (11) |  | 19 (13) |  |  |  |  |  |
| **Etiology** |  |  |  |  | 0.062 |  |  |  |
| HBV | 45(83.3) |  | 128(90.8) |  |  |  |  |  |
| HCV | 2(3.7) |  | 0(0.0) |  |  |  |  | 0.999 |
| HBV+HCV | 0(0.0) |  | 0(0.0) |  |  |  |  |  |
| Other | 7(13.0) |  | 13(9.2) |  |  |  |  | 0.394 |
| **NE (10^9^/L)** | 3.28 (2.69-4.11) |  | 3.77 (2.96-4.76) |  | 0.028 |  | 0.72 (0.55-0.92) | 0.014 |
| **HBV-DNA (IU/ml)** | 1.08×10^4^ (0.00-5.35×10^5^) |  | 2.49×10^3^(0.00-3.06×10^5^) |  | 0.320 |  |  | 0.127 |
| **AFP (ng/ml)** |  |  |  |  | 0.060 |  | 2.12 (0.97-4.56) | 0.056 |
| ≤ 400 | 40 (74) |  | 121 (86) |  |  |  |  |  |
| >400 | 14 (26) |  | 20 (14) |  |  |  |  |  |
| **ALT (U/L)** |  |  |  |  | 0.480 |  |  | 0.379 |
| ≤ 50 | 36 (67) |  | 103 (73) |  |  |  |  |  |
| >50 | 18 (33) |  | 38 (67) |  |  |  |  |  |
| **AST (U/L)** |  |  |  |  | 0.001 |  | 3.16 (1.63-6.18) | 0.001 |
| ≤ 40 | 28 (52) |  | 109 (73) |  |  |  |  |  |
| >40 | 26 (48) |  | 32 (23) |  |  |  |  |  |
| **GGT (U/L)** |  |  |  |  | 0.005 |  | 2.50 (1.32-4.78) | 0.005 |
| ≤ 60 | 24 (44) |  | 94 (67) |  |  |  |  |  |
| >60 | 30 (56) |  | 47 (33) |  |  |  |  |  |
| **BCLC stage** |  |  |  |  | 0.027 |  |  |  |
| 0 | 5(9.3) |  | 38(27.0) |  |  |  |  |  |
| A | 41(75.9) |  | 89(63.1) |  |  |  | 3.50(1.28-9.55) | 0.014 |
| B | 8(14.8) |  | 14(9.9) |  |  |  | 4.34(1.21-15.53) | 0.024 |
| **Tumor size (mm)** |  |  |  |  | 0.336 |  |  | 0.282 |
| ≤ 30 | 26 (48) |  | 80 (57) |  |  |  |  |  |
| >30 | 28 (52) |  | 61 (43) |  |  |  |  |  |
| **AP enhancement type** |  |  |  |  | 0.610 |  |  | 0.586 |
| Type 1 | 1 (2) |  | 7 (5) |  |  |  |  |  |
| Type 2 | 9 (17) |  | 34 (24) |  |  |  |  |  |
| Type 3 | 35 (65) |  | 77 (55) |  |  |  |  |  |
| Type 4 | 5 (9) |  | 10 (7) |  |  |  |  |  |
| Type 5 | 4 (7) |  | 13 (9) |  |  |  |  |  |
| **Capsule appearance** |  |  |  |  | 0.898 |  |  | 0.971 |
| Absent | 19 (35) |  | 50 (36) |  |  |  |  |  |
| Incomplete | 12 (22) |  | 35 (25) |  |  |  |  |  |
| Complete | 23 (43) |  | 56 (40) |  |  |  |  |  |
| **Hypodense halo** |  |  |  |  | 0.451 |  |  | 0.421 |
| Absent | 50 (93) |  | 125 (89) |  |  |  |  |  |
| Present | 4 (7) |  | 16 (11) |  |  |  |  |  |
| **Intratumor necrosis** |  |  |  |  | 0.723 |  |  | 0.708 |
| Absent | 38 (70) |  | 103 (73) |  |  |  |  |  |
| Present | 16 (30) |  | 38 (27) |  |  |  |  |  |
| **Satellite nodule** |  |  |  |  | 0.181 |  |  | 0.186 |
| Absent | 49 (91) |  | 135 (96) |  |  |  |  |  |
| Present | 5 (9) |  | 6 (4) |  |  |  |  |  |
| **Peritumoral hypointensity** |  |  |  |  | 0.176 |  |  | 0.146 |
| Absent | 46 (85) |  | 130 (92) |  |  |  |  |  |
| Present | 8 (15) |  | 11 (8) |  |  |  |  |  |
| **MVI** |  |  |  |  | <0.001 |  | 3.92 (2.00-7.80) | <0.0001 |
| Absent | 28 (52) |  | 114 (81) |  |  |  |  |  |
| Present | 26 (48) |  | 27 (19) |  |  |  |  |  |
| **Tumor number** |  |  |  |  | 0.043 |  | 1.68 (1.01-2.81) | 0.043 |
| 1 | 38 (70) |  | 117 (83) |  |  |  |  |  |
| 2 | 11 (20) |  | 19 (13) |  |  |  |  |  |
| 3 | 4 (7) |  | 4 (3) |  |  |  |  |  |
| 4 | 1 (2) |  | 1 (1) |  |  |  |  |  |
| **Histologic grade** |  |  |  |  | 0.039 |  |  |  |
| well differentiated | 1 (1.9) |  | 20 (14.2) |  |  |  |  |  |
| moderately differentiated | 31 (57.4) |  | 76 (53.9) |  |  |  | 8.158 (1.049-63.455) | 0.045 |
| poorly differentiated | 22 (40.7) |  | 45 (31.9) |  |  |  | 9.778 (1.231-77.648) | 0.031 |

**Note:** Data are presented as number (%) or median (interquartile range, IQR). OR, odds ratio; CI, confidence interval; HBV, hepatitis B virus; HCV, hepatitis C virus; NE, neutrophil count; AFP, alpha-fetoprotein; ALT, alanine amino-transferase; AST, aspartate amino-transferase; GGT, γ-glutamyl transpeptadase; BCLC, barcelona clinic liver cancer; AP, arterial phase; Type 1, a homogeneous enhancement pattern with no increased arterial blood flow; Type 2, a homogeneous enhancement with increased arterial blood flow; Type 3, a heterogeneous enhancement included non-enhanced areas; Type 4, a heterogeneous enhancement pattern with irregular ring-like structures; Type 5, a heterogeneous and hypointense enhancement pattern; MVI, microvascular invasion.

**Supplementary Table 5. Intra- and inter- readers agreement of MR image features**

| **Image features** | **Reader 1**  **(n=50)** | **Reader1_intra**  **(n=50)** | **Reader 2**  **(n=50)** | **Reader 3**  **(n=50)** | **κ_R1** | **κ_AR** |
| --- | --- | --- | --- | --- | --- | --- |
| **Tumor size(mm)** |  |  |  |  | 0.92 | 0.97 |
| ≤30 | 27 (54) | 27 (54) | 28 (56) | 28 (56) |  |  |
| ＞30 | 23 (46) | 23 (46) | 22 (44) | 22 (44) |  |  |
| **AP enhancement type** |  |  |  |  | 0.84 | 0.84 |
| Type 1 | 1 (2) | 1(2) | 2(4) | 2(4) |  |  |
| Type 2 | 12 (24) | 11(22) | 13(26) | 13(26) |  |  |
| Type 3 | 27 (54) | 30(60) | 26(52) | 25(50) |  |  |
| Type 4 | 5 (10) | 3(6) | 5(10) | 6(12) |  |  |
| Type 5 | 5 (10) | 5(10) | 4(8) | 4(8) |  |  |
| **Capsule appearance** |  |  |  |  | 0.95 | 0.78 |
| Absent | 16 (32) | 16(32) | 15(30) | 13(26) |  |  |
| Incomplete | 12(24) | 12(24) | 12(24) | 18(36) |  |  |
| Complete | 22(44) | 22(44) | 23(46) | 19(38) |  |  |
| **Hypodense halo** |  |  |  |  | 0.81 | 0.72 |
| Absent | 43(86) | 45(90) | 42(84) | 39(78) |  |  |
| Present | 7(14) | 5(10) | 8(16) | 11(22) |  |  |
| **Intratumor necrosis** |  |  |  |  | 0.82 | 0.79 |
| Absent | 39(78) | 40(80) | 36(72) | 38(76) |  |  |
| Present | 11(22) | 10(20) | 14(28) | 12(24) |  |  |
| **Satellite nodules** |  |  |  |  | 1.00 | 0.74 |
| Absent | 48(96) | 48(96) | 49(98) | 49(98) |  |  |
| Present | 2(4) | 2(4) | 1(2) | 1(2) |  |  |
| **Peritumoral hypointensity** |  |  |  |  | 0.85 | 0.75 |
| Absent | 47(94) | 46(92) | 48(96) | 47(94) |  |  |
| Present | 3(6) | 4(8) | 2(4) | 3(6) |  |  |

**Note:** Data are presented as number (%). κ_R1, the Cohen κ coefficient value of reader 1. κ_AR, the Fleiss κ statistic of interreader agreement for all three readers. AR, all readers. AP, arterial phase; Type 1, a homogeneous enhancement pattern with no increased arterial blood flow; Type 2, a homogeneous enhancement with increased arterial blood flow; Type 3, a heterogeneous enhancement included non-enhanced areas; Type 4, a heterogeneous enhancement pattern with irregular ring-like structures; Type 5, a heterogeneous and hypointense enhancement pattern.

**Supplementary Table 6. Specific follow-up of early recurrence cases**

| **Set** | **RFS**  **(months)** | **Intrahepatic recurrence** | | | | **Extrahepatic recurrence** | | **Concurrent intra- and extrahepatic recurrence** |
| --- | --- | --- | --- | --- | --- | --- | --- | --- |
|  |  | **Typical CT/MR findings** | **Atypical CT/MR findings** | | | **Typical CT/MR findings** | **Histological analysis** |  |
|  |  |  | **Biopsy**  **confirmed HCC** | **Re-postoperative confirmed HCC** | **TACE staining** |  |  |  |
| **Training**  (n=54) | 8.5  (4.0-13.0) | 32 (59.3) | 0 | 2 (3.7) | 1 (1.9) | 20 (3.7) | 2 (3.7) | 5 (9.2) |
| **Validation** (n=23) | 8.0  (3.0-13.0) | 16 (69.6) | 0 | 1 (4.3) | 0 | 5 (21.7) | 2 (8.7) | 0 |
| **Total**  (n=77) | 8.0  (4.0-13.0) | 48 (62.3) | 0 | 3 (3.9) | 1 (1.3) | 25 (32.5) | 4 (5.2) | 5 (6.5) |

**Note:** Data are presented as number (%) or median (interquartile range, IQR). RFS, recurrence-free survival; CT, computed tomography; MR, magnetic resonance; HCC, hepatocellular carcinoma; TACE, transarterial chemoembolization.
